# Day-Level Mediators of Change in Brief Motivational Intervention for Alcohol Use and Liver Health

**DOI:** 10.21203/rs.3.rs-9943945/v1

**Published:** 2026-06-10

**Authors:** Abhishek Aggarwal, Peter M. Monti, Molly Magill, Kittichai Promrat, Jessica L. Mellinger, Hayley Treloar Padovano

**Affiliations:** Brown University; Brown University; Brown University; Brown University Health; Henry Ford Health; Brown University

**Keywords:** alcohol use disorder, brief motivational intervention, biobehavioral feedback, daily diary survey, multilevel mediation

## Abstract

**Background:**

Day-level motivation to limit drinking is theorized to be a proximal mechanism of change in motivational interviewing (MI) interventions targeting alcohol intake, but day-level evidence remains limited. This study examined whether brief MI with personalized biobehavioral feedback was associated with changes in daily drinking among adults with alcohol use disorder (AUD) and alcohol-associated liver disease (ALD), and whether day-level importance and confidence to limit drinking served as within-person mediators of change.

**Methods:**

Participants (*N =* 33; *M* age = 46.0 (12.55); day-level observations = 799) completed morning surveys for 28 days, including up to 10 pre-intervention days. Multilevel interrupted time-series models partitioned intervention effects into pre-intervention slope, intervention-day level shift, and post-intervention slope, and tested whether the intervention’s effects on daily drinking were mediated by day-level importance and confidence to limit drinking.

**Results:**

Daily drinking outcomes declined during the pre-intervention period, with no significant intervention-day shifts or post-intervention trends. However, the intervention-day shift was associated with increased day-level importance of limiting drinking, and higher-than-usual importance and confidence in limiting drinking on a given day were associated with fewer planned and actual drinks and lower odds of any drinking and heavy drinking. The intervention-day shift exerted significant indirect effects on daily drinking outcomes through same-day increases in importance of limiting drinking.

**Conclusions:**

Intensive longitudinal data supported perceived importance of limiting drinking as a proximal day-level mediator of change in brief AUD/ALD interventions, suggesting that frequent, day-level reinforcement of the personal salience of drinking reduction may be a key intervention target.

## 1. Introduction

Alcohol use disorder (AUD) remains highly prevalent and undertreated in the United States, with 28.9 million individuals meeting past-year criteria in 2023 and fewer than one in ten receiving treatment (Substance Abuse and Mental Health Services Administration, 2024). Alcohol-associated liver disease (ALD) is one of the most severe medical consequences of AUD, contributing to approximately one-quarter of cirrhosis-related deaths (Anouti & Mellinger, 2023; Pan et al., 2025). Although clinical guidance recommends routine alcohol screening, brief intervention, and referral to treatment for AUD in primary and secondary care settings (Crabb et al., 2020), a critical gap remains in integrating alcohol-focused behavioral interventions into the continuum of care for adults with AUD who have, or are at risk for, ALD. Translating brief motivational interviewing (MI; a well-established behavioral intervention for AUD) into integrated clinical settings is a promising approach to optimize care for patients with AUD and ALD.

Meta-analyses show that brief MI, sometimes in a single session, reduces alcohol consumption relative to no treatment, with the clearest effects on drinking quantity at follow-ups of three months or less (Vasilaki et al., 2006; Mun et al., 2023; Tan et al., 2023). While these standalone effects tend to fade over time, pairing MI with personalized normative feedback outperforms either component alone (Carey et al., 2007; Walters et al., 2009). Building on this evidence, biobehavioral feedback that makes the physical consequences of alcohol use more salient, such as biomarker-anchored summaries of liver health, is increasingly investigated as a clinically meaningful intervention lever in hepatology (Mellinger et al., 2023; Treloar Padovano et al., 2026). As personalized interventions continue to be tested, understanding the proximal mechanisms driving *in vivo* drinking reductions (e.g., through shifting day-to-day motivational components) provides key empirical insights required to directly guide intervention development and refinement.

Motivational shifts in MI are indicated by the growth of change talk (i.e., patient statements favoring behavior change) versus sustain talk (i.e., statements favoring maintaining the status quo). The active ingredients of MI are commonly understood as comprising two complementary components: a relational, non-directive component centered on therapist empathy, alliance, and avoidance of MI-inconsistent behaviors, and a technical, directional component that evokes and reinforces client change talk while reducing sustain talk (Miller & Rose, 2009; Apodaca & Longabaugh, 2009). The technical component is theorized to evoke and strengthen change talk through two within-person processes: motivation (in-session statements about desires, reasons, need, and commitment to change) and self-efficacy (statements about ability and confidence to change) (Amrhein, 2004; DiClemente et al., 2004). In this regard, a proficient MI session should not only strengthen in-session motivation and self-efficacy but also translate into real-world, day-to-day changes in these processes that support lasting reductions in drinking. While converging meta-analytic results show that in-session verbalizations toward change influence MI efficacy, characterizing the real-world motivational and self-efficacy processes captures their proximal ecological effects in the days following an MI intervention.

Though often assessed as static, person-level indicators, theories of behavior change conceptualize motivation (i.e., importance of change) and self-efficacy (i.e., confidence to change) as dynamic states that fluctuate over time in response to real-world contexts (Bandura, 1982; Brown, 1998). Daily-diary designs provide direct *in vivo* measurement of these key constructs as they unfold in daily life. Prior daily-diary work provides novel evidence that motivation and self-efficacy predict drinking outcomes during treatment among adults with drinking-reduction goals (Kuerbis et al., 2013) and that within-person fluctuations in these constructs predict next-day drinking (Morgenstern et al., 2016). Building on this work, Kuerbis et al. (2019) found that daily-diary-assessed commitment and confidence predicted day-level drinking among adult problem drinkers, although directional MI did not strengthen these links relative to a relational-only condition or a non-therapy control. Together, these studies suggest that daily importance and confidence may be clinically meaningful change processes for drinking outcomes following MI interventions, but whether they operate as day-level mechanisms of change in personalized biobehavioral MI interventions for adults with AUD, including those with ALD, remains unknown.

### 1.1 Study Aims

The present study addresses this gap using 28 days of morning daily diary data from 33 adults with AUD/ALD who completed a brief MI session enhanced with personalized biobehavioral feedback. We examined whether the intervention increased day-level importance and confidence to limit drinking and whether higher-than-usual importance or confidence on a given day mediated four daily drinking outcomes: number of standard drinks, any drinking day, heavy drinking day, and planned drinks for the day ahead. Intervention exposure was modeled using a piecewise interrupted time-series specification that included a pre-intervention slope, an intervention-day level shift, and a post-intervention slope (Lopez Bernal et al., 2017; Wagner et al., 2002). Importance and confidence were entered simultaneously in the model, allowing each construct’s association with daily drinking to be estimated above and beyond the other (MacKinnon, 2008; Preacher & Hayes, 2008). We hypothesized that the intervention would increase day-level importance and confidence to limit drinking and that higher-than-usual values on these constructs on a given day would predict lower drinking and fewer planned drinks that day.

## 2. Methods

### 2.1. Participants and Procedures

The analytic sample comprised 33 adults with AUD, including 5 with ALD, who completed a brief MI session enhanced with personalized biobehavioral feedback as part of a nonrandomized, parallel-assignment intervention study. Participants provided up to 28 days of morning daily diary data, including a pre-intervention monitoring phase of up to 10 days, followed by baseline questionnaires, including the Alcohol Use Disorders Identification Test (AUDIT), a 60-minute MI session, and 18 additional days of self-monitoring. Morning diary surveys were delivered via a custom smartphone app (MetricWire, Inc., 2023), triggered silently at 4:00 a.m. local time and completed upon waking. Up to three reminders per prompt (push notification, text, automated call) were sent to maximize compliance.

MI session fidelity was high based on the Motivational Interviewing Treatment Integrity (MITI) 4.2.1 system (Moyers et al., 2016). Full eligibility, recruitment, intervention fidelity, and primary outcome procedures are reported elsewhere (Treloar Padovano et al., in review). The study was preregistered at ClinicalTrials.gov (NCT05135767) and approved by the Brown University Institutional Review Board (IRB; 2011002840), with Lifespan IRB (1712272) serving as a relying site. All participants provided written informed consent before enrollment.

### 2.2. Daily Measures

#### 2.2.1. Standard drinks consumed.

At each morning prompt, participants reported prior-day alcohol use using a free-response numeric item: “*How many STANDARD DRINKS of ALCOHOL did you have YESTERDAY?*” Participants entered 0 if they did not drink any alcohol.

#### 2.2.2. Any drinking and heavy drinking.

Two binary drinking indicators were derived from the daily standard drinks count in Section 2.2.1. Any drinking was coded as 1 for any non-zero standard drink count and 0 otherwise. Heavy drinking day was coded 1 when daily drinks met NIAAA sex-specific thresholds: ≥4 drinks for women or ≥5 for men, and 0 otherwise (National Institute on Alcohol Abuse and Alcoholism, 2023).

#### 2.2.3. Planned drinks.

Each morning prompt also assessed planned drinking for the day ahead using a free-response numeric item, “*How many STANDARD DRINKS of ALCOHOL do you PLAN to drink TODAY?*”

#### 2.2.4. Importance of limiting drinking.

At each morning prompt, participants reported the day-level importance of limiting drinking using the standard motivational interviewing importance ruler (Miller & Rollnick, 2013). Response options ranged from 0 = Not at all to 10 = Extremely: “*How IMPORTANT is it for you to AVOID or LIMIT DRINKING TODAY?*”

#### 2.2.5. Confidence in limiting drinking.

Day-level confidence in limiting drinking was assessed at each morning prompt using the parallel motivational interviewing confidence ruler (Miller & Rollnick, 2013). Response options ranged from 0 = Not at all to 10 = Extremely: “*How CONFIDENT are you in your ability to AVOID or LIMIT DRINKING TODAY?*”

#### 2.2.6. Intervention day-level shift and piecewise time variables.

Intervention day-level shift was coded 0 for diary days on or before the intervention and 1 for post-intervention days. Two piecewise time variables modeled an interrupted time series with a knot at each participant’s intervention day (Lopez Bernal et al., 2017; Wagner et al., 2002). The pre-intervention slope increased from −10 to 0 and remained 0 thereafter. The post-intervention slope was 0 through intervention day and increased linearly afterward.

### 2.3. Covariates

All multilevel models adjusted for age (centered), gender (1 = male, 0 = female), education (1 = *some high school* through 6 = *graduate degree*), ethnicity (1 = Hispanic, 0 = non-Hispanic), financial status (1 = *cannot make ends meet* through 4 = *well-off*), baseline AUDIT score (Saunders et al., 1993; sample centered), and weekend (1 = Saturday/Sunday, 0 = weekday). To disentangle and isolate day-level mediation effects, person-level aggregated means (sample centered) were created from daily measures of importance and confidence and included as covariates.

### 2.4. Data Preprocessing

The daily diary survey assessed the number of drinks consumed on the previous day; therefore, this outcome was forward-lagged by one calendar day to align it with the planning, importance, and confidence ratings reported for that same day. Similarly, the binary indicators, including any drinking and heavy drinking day, were also shifted. Additionally, each mediator (importance, confidence) was decomposed by person-mean centering: the within-person component captured each day’s deviation from the participant’s own mean, and the between-person component captured the grand-mean-centered participant mean (Enders & Tofighi, 2007). After listwise deletion of records missing any model variable, regression models were fit using 799 person-days.

### 2.5. Statistical Analysis

Mediation of intervention-related changes in daily drinking by day-level importance and confidence was tested within a 1-1-1 multilevel mediation framework (Bauer et al., 2006; Preacher et al., 2010), with the total effect, a-paths, and b-paths estimated separately. The total-effect model (Table 2) regressed each outcome on the intervention-day level shift, pre-intervention slope, and post-intervention slope; two a-path models regressed each mediator (importance, confidence) on the same intervention predictors (Table 3a); and b-path models regressed each outcome on the within- and between-person components of both mediators entered simultaneously to estimate each mediator’s unique effect, while retaining the intervention predictors to yield the residual direct effect (Table 3b). All models adjusted for covariates.

All analyses were conducted in R version 4.3.3. Total-effect and b-path models (drinking outcomes) were fit using glmmTMB (Brooks et al., 2017): count outcomes (daily standard drinks, daily planned drinks) used a negative binomial type 2 distribution with log link (Atkins et al., 2013), and binary outcomes (any drinking day, heavy drinking day) used a Bernoulli distribution with logit link. A-path models (importance, confidence) were fit as linear mixed models in lme4 (Bates et al., 2015b) with lmerTest (Kuznetsova et al., 2017) for Satterthwaite-approximated degrees of freedom. All models included a random intercept for participant (Bolger & Laurenceau, 2013). Random-slope structures were evaluated against random-intercept-only specifications via likelihood-ratio tests; full random-effects selection details are reported in Supplementary File A(Bates et al., 2015a; Matuschek et al., 2017).

Indirect effects were defined as the product of the a-path and b-path on the link (log or logit) scale (MacKinnon, 2008). For each indirect effect, 95% confidence intervals were estimated using a Monte Carlo method with 20,000 simulation draws (MacKinnon et al., 2004; Preacher & Selig, 2012), with a- and b-path estimates sampled from normal distributions parameterized by model-estimated coefficients and standard errors. As the a- and b-paths were estimated in separate models, their cross-equation covariance could not be directly estimated; therefore, the two paths were sampled independently (Preacher & Selig, 2012).

Two sensitivity analyses assessed the robustness of the indirect-effect inference. First, leave-one-participant-out analyses refit the a- and b-path models excluding one participant at a time to examine whether any single participant disproportionately influenced the indirect effects (Nieuwenhuis et al., 2012). Second, profile-likelihood confidence intervals were computed for the a-path coefficients as a small-sample robustness check on Wald-based inference (Bates et al., 2015b; Bolker et al., 2009). The indirect-effect inference was robust in both analyses. Full results are reported in Supplementary File B.

## 3. Results

### 3.1. Demographics and Descriptive Statistics

Descriptive statistics are presented in Table 1. The analytic sample included 33 adults with AUD, including 5 with ALD (*M* age = 46.0, *SD* = 12.55; 51.5% male; 30.3% Hispanic), who contributed 799 person-days of daily diary data, of which 31.2% were pre-intervention and 68.8% were post-intervention. Participants reported a mean of 2.73 drinks per day (SD = 3.52); 57.3% of days involved any drinking, and 28.0% met heavy drinking criteria. Planned drinking for the same day averaged 1.75 drinks (*SD* = 2.66). Day-level importance of limiting drinking averaged 5.01 (*SD* = 3.84) and confidence to limit drinking averaged 5.75 (*SD* = 3.22), both on 0–10 scales. Intraclass correlation coefficients indicated substantial between-person variance in importance (ICC = .70) and confidence (ICC = .49). The between-person correlation of importance and confidence was *r* = .45 (95% CI [.13, .69], *p* = .008), and the within-person correlation was *r* = .53 (95% CI [.48, .58], *p* < .001).

### 3.2. Total Effects of the Intervention on Daily Drinking Outcomes

Unadjusted total-effect estimates without mediators are presented in Table 2. The intervention-day level shift and post-intervention slope were not significantly associated with any drinking outcome; however, the pre-intervention slope was negatively associated with all four outcomes. Specifically, each additional pre-intervention day was associated with lower expected drinks/day (IRR = 0.90, 95% CI [0.85, 0.95], *p* < .001), lower odds of any drinking on that day (OR = 0.86, [0.77, 0.96], *p* = .007), lower odds of a heavy drinking day (OR = 0.84, [0.74, 0.95], *p* = .006), and fewer planned drinks (IRR = 0.94, [0.89, 0.99], *p* = .032).

### 3.3. Mediation by Importance and Confidence of Limiting Drinking

#### 3.3.1. A-paths (Table 3a).

The intervention-day level shift was associated with a significant increase in day-level importance of limiting drinking (*b* = 0.73, *SE* = 0.29, *p* = .011), but not confidence. Rather, daily confidence showed a positive pre-intervention slope (*b* = 0.14, *SE* = 0.05, *p* = .008). Neither importance or confidence showed a significant post-intervention slope change.

#### 3.3.2. B-paths (Table 3b).

After adjusting for the interrupted time-series intervention effects and other covariates, higher-than-usual importance of limiting drinking on a given day was associated with fewer drinks (IRR = 0.92, 95% CI [0.87, 0.97], *p* = .001), lower odds of any drinking (OR = 0.83, [0.75, 0.93], *p* < .001), lower odds of heavy drinking (OR = 0.78, [0.64, 0.96], *p* = .018), and fewer planned drinks (IRR = 0.83, [0.78, 0.89], *p* < .001) on that day. Higher-than-usual confidence in limiting drinking showed a similar pattern of negative associations across all outcomes (IRRs/ORs 0.68 to 0.88, all *p* < .001). At the person level, a participant’s average confidence (sample centered) was also negatively associated with all four drinking outcomes, whereas person-level **importance** (sample centered) was associated only with fewer planned drinks.

#### 3.3.3. Indirect effects (Table 3c; Figure 1).

The intervention-day level shift exerted a significant indirect effect on every outcome through within-person importance of limiting drinking (drinks/day: indirect = −0.064, 95% CI [−0.138, −0.011], *p* = .010; any drinking: −0.132, [−0.282, −0.023], *p* = .012; heavy drinking day: −0.181, [−0.430, −0.012], *p* = .028; planned drinks: −0.136, [−0.260, −0.029], *p* = .009). The indirect path through confidence was not significant for any outcome.

## 4. Discussion

This study examined whether a brief MI session targeting alcohol use and liver health, enhanced with personalized biobehavioral feedback, was associated with day-level changes in drinking among adults with AUD, and whether within-person importance and confidence to limit drinking served as mediators of change. Using a multilevel interrupted time-series framework that separated pre-intervention trends, the intervention-day level shift, and post-intervention trends, total-effect models showed pre-intervention declines in all four daily drinking outcomes, without additional significant intervention-day level shifts or significant post-intervention slope changes. Intervention-day level shift increased day-level importance of limiting drinking, but not confidence (path *a*), while both within-person importance and confidence were uniquely associated with improved drinking outcomes (path *b*) after controlling for the interrupted time series intervention effects. The intervention-day level shift exerted significant indirect effects on every drinking outcome through improved within-person importance of limiting drinking, but not confidence. Together, these findings provide a real-world, day-level demonstration of perceived importance of limiting drinking as a proximal mediator of change following brief MI, indicating that frequent, day-level reinforcement of the personal salience of drinking reduction may be a key intervention target for sustaining behavior change.

### 4.1. Anticipatory (Pre-Intervention) Drinking Reductions

Pre-intervention slope effects were significant for all four drinking metrics. Specifically, drinking odds and amounts (on drinking days) declined leading up to the intervention. At least two non-mutually exclusive processes may have contributed to these declines. First, the pre-intervention lead-in period including daily reports of mediators (importance confidence) and standard drinks consumed and planned drinking, together with validated interviewer-administered alcohol assessments, may itself have acted as an intervention through a self-monitoring mechanism (Clifford et al., 2007; Clifford & Davis, 2012). For example, hazardous drinkers who monitored alcohol use daily through a smartphone app reduced their intake by approximately 0.80 standard drinks over 21 days, whereas non-hazardous drinkers showed no change (Poulton et al., 2019). Second, anticipation of an upcoming intervention may itself shift drinking (Stasiewicz et al., 2013; Worden et al., 2008), and participants in the present study knew that a brief MI session was scheduled within the first two weeks of enrollment. For example, Epstein et al. (2005) found that 44% of 102 alcohol-dependent women enrolled in a clinical trial of behavioral treatment became abstinent during the pretreatment assessment period before any treatment was delivered. Thus, self-monitoring and treatment anticipation may explain the pre-intervention declines.

The non-significant intervention-day level shift and post-intervention slope are also consistent with evidence that brief MI for AUD may not always produce acute drinking reductions beyond those associated with active assessment, feedback, or other nonspecific intervention components (Kaner et al., 2018; Morgenstern et al., 2017; Vasilaki et al., 2006). The current findings can also co-exist with the primary outcomes of the parent trial, which found significant overall pre- to post-intervention drinking reductions (Treloar Padovano et al., in review). The parent trial examined broader three-month drinking windows, whereas the present analysis isolated proximal day-level changes surrounding the intervention. Importantly, the absence of significant direct intervention effects does not preclude indirect effects, as the intervention may have influenced drinking through more proximal motivational processes.

### 4.2. Significant Intervention Effects On Importance But Not Confidence (a-paths)

The intervention-day level shift was associated with an increase in day-level importance of limiting drinking but not in confidence, whereas confidence showed a positive pre-intervention slope. Two complementary interpretations may explain this pattern. First, importance and confidence are theoretically distinct constructs in MI: importance reflects why a person should change, whereas confidence reflects whether they believe they can change (Miller & Rollnick, 2013; Rollnick et al., 1992). Personalized biobehavioral feedback, particularly the liver-health information provided by results of liver function blood tests, provides an objective, personalized marker of harm that maps directly onto perceived severity and personal salience of change. Early evidence is consistent with this mapping: transient elastography results increased readiness for change among inpatients with AUD (Rutledge et al., 2025), and patients have described serial FibroScan values as a key motivational anchor (Rennick-Egglestone et al., 2023; Subhani et al., 2021). In contrast, biomarker feedback does not provide the mastery experiences, vicarious learning, or skills rehearsal that are the established sources of self-efficacy (Bandura, 1982). The positive pre-intervention slope for confidence may instead reflect an early self-monitoring effect, whereby daily tracking of alcohol use fosters a growing sense of self-efficacy over drinking behavior. The absence of a comparable pre-intervention increase in importance supports the proposed distinction between the two constructs: importance may be more responsive to personalized biomarker feedback about alcohol-related harm than to self-monitoring alone.

### 4.3. Within- And Between-Person Associations Of Importance And Confidence With Drinking (b-paths)

Higher-than-usual importance and confidence on a given day each uniquely predicted lower drinking on those days, including fewer drinks, lower odds of any or heavy drinking, and fewer planned drinks. This finding replicates and extends prior daily-diary work in problem drinkers showing that morning reports of motivation and self-efficacy operate as unique within-person predictors of subsequent drinking (Kuerbis et al., 2013; Morgenstern et al., 2016), and is consistent with theoretical frameworks treating motivation and self-efficacy as parallel, non-redundant processes involved in resolving ambivalence (Miller & Rose, 2009). At the between-person level, confidence was negatively associated with all four outcomes, indicating that participants with higher average confidence showed more favorable patterns of actual and planned drinking, whereas between-person importance was associated only with fewer planned drinks. Together, this pattern indicates that confidence predicts drinking at both between- and within-person levels, whereas importance predicts drinking primarily within-person. Clinically, this distinction implies that reinforcing the day-to-day salience of drinking reduction may be especially useful through frequent within-person prompts, whereas confidence may benefit from a combination of frequent prompts and broader, trait-level interventions, such as structured skills training and mastery experiences.

### 4.4. Indirect Effects and Clinical Implications

The intervention-day level shift exerted significant indirect effects on every drinking outcome through within-person importance, but not through confidence, primarily because the intervention did not shift confidence itself (non-significant a-path). Thus, while confidence remained a meaningful day-level predictor of drinking outcomes, it was not shaped by the MI intervention. Confidence therefore remains a viable intervention target even though it was not the active mechanism in the present brief intervention. The current findings support augmenting brief MI for AUD/ALD with frequent, ongoing day-level support that reinforces personal salience of drinking reduction. As confidence operated on drinking independently of the intervention, parallel prompts targeting confidence, including brief skills rehearsal or mastery cue prompts, may extend day-level benefit. Building on these findings, future just-in-time adaptive interventions (JITAIs; Nahum-Shani et al., 2018) for AUD/ALD could test whether days with lower-than-usual confidence or importance serve as states of vulnerability that warrant tailored just-in-time support.

### 4.5. Limitations and Future Directions

Several limitations warrant consideration. First, although the 28-day assessment period provided rich intensive longitudinal data, the sample included 33 adults with AUD/ALD from a single site, limiting generalizability. Second, MI and personalized biobehavioral feedback were delivered as a single intervention, so the present design cannot isolate which component increased the day-level importance of limiting drinking. Future multi-arm dismantling or factorial designs comparing brief MI alone, biobehavioral feedback alone, MI with feedback, and a self-monitoring-only control would clarify whether the importance pathway identified here is specifically driven by objective biomarker feedback. Third, given the modest person-sample size, we did not estimate a fully integrated multilevel structural equation model in which the a- and b-paths are jointly estimated (Preacher et al., 2010). However, the current approach, i.e., estimating the a- and b-paths separately and computing indirect effects using Monte Carlo simulation, is appropriate for the present sample size and a relative strength of this approach which reduces bias over uncorrected or untested (assumed) mechanistic effects. Fourth, the within-person mediator-outcome associations (b-paths) were estimated by pooling all available person-days rather than the post-intervention phase alone, even though these associations were weighted toward the post-intervention period (~ 69% post-intervention observations). Larger studies with longer follow-up should test whether this association holds specifically after the intervention.

## 5. Conclusion

This study examined day-level mediators of change in a brief MI session enhanced with personalized biobehavioral feedback among adults with AUD/ALD. Total-effect models demonstrated clear pre-intervention declines in all four daily drinking outcomes, without additional significant movement in drinking metrics thereafter. Nonetheless, the intervention-day level shift increased day-level importance of limiting drinking, though not confidence, and within-person importance and confidence each uniquely predicted lower drinking across the study period. Notably, the intervention-day level shift exerted significant indirect effects on every drinking outcome through within-person importance, but not confidence. Together, these findings identify perceived importance of limiting drinking as a proximal day-level mediator of change following brief MI in AUD. Frequent, day-level reinforcement of the personal salience of drinking reduction, alongside complementary prompts targeting confidence, may be a promising direction for next-generation interventions designed to sustain behavior change beyond the MI session itself.

## Supplementary Material

This is a list of supplementary files associated with this preprint. Click to download.

SupplementaryFileA.docx

SupplementaryFileB.docx

## Figures and Tables

**Figure 1:**
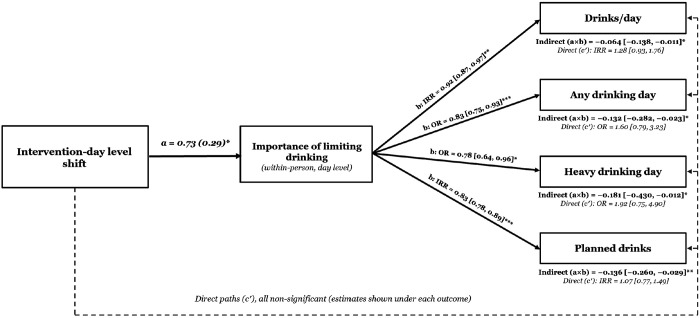
Day-Level Importance of Limiting Drinking as a Mediator of Brief MI Effects on Daily Drinking Outcomes *Note.* Solid paths are statistically significant; dashed paths are non-significant. Path a: unstandardized coefficient (SE) from a linear mixed-effects model. Paths b and c′: incidence-rate ratios (IRR) or odds ratios (OR) [95% CI] from negative binomial or logistic mixed-effects models, adjusted for pre- and post-intervention slopes, covariates, and person-level mediator means. Indirect effects = a × b on the link scale, with Monte Carlo 95% CIs (20,000 draws). Confidence in limiting drinking was modeled simultaneously but had no significant a-path or indirect effects. *p < .05. **p < .01. ***p < .001.

**Table 1:** Demographics and Descriptive Statistics (N = 33; Observations = 799)

Variable	n / Mean	% / SD
*Daily outcomes and mediators (Observations = 799)*		
Number of drinks/day (0–25)	2.73	3.52
Any drinking day		
No (0)	341	42.7%
Yes (1)	458	57.3%
Heavy drinking day		
No (0)	575	72.0%
Yes (1)	224	28.0%
Planned drinks today (0–19)	1.75	2.66
Importance of limiting drinking today (0–10)	5.01	3.84
Confidence in limiting drinking today (0–10)	5.75	3.22
Intervention-day level shift		
Pre (0)	249	31.2%
Post (1)	550	68.8%
*Person-level control variables (N = 33)*		
Age (years)	46.00	12.55
Gender		
Female	16	48.5%
Male	17	51.5%
Education (Last Grade/Year Completed)		
1 = Some high school	1	3.0%
2 = High school graduate / GED	2	6.1%
3 = Some college / vocational	10	30.3%
4 = Associate / Bachelor’s	8	24.2%
5 = Some graduate school	4	12.1%
6 = Graduate degree	8	24.2%
Ethnicity		
Non-Hispanic	23	69.7%
Hispanic	10	30.3%
Financial Status		
1 = Cannot make ends meet	9	27.3%
2 = Just enough to get by	10	30.3%
3 = Comfortable	11	33.3%
4 = Well-off	3	9.1%
AUDIT total score	17.09	8.59
*Within-person control variables (Observations = 799)*		
Weekend		
No (0)	572	71.6%
Yes (1)	227	28.4%

*Note*. Heavy drinking day is defined as ≥ 4 standard drinks for women and ≥ 5 standard drinks for men (NIAAA criterion).

**Table 2:** Total Effects of the Intervention on Daily Drinking Outcomes (N = 33 participants, Observations = 799 person-days)

Predictor	Drinks/day	Any drinking	Heavy drinking day	Planned drinks
	*IRR [95% CI]*	*OR [95% CI]*	*OR [95% CI]*	*IRR [95% CI]*
*Time and intervention effects*				
Pre-intervention slope	0.90 [0.85, 0.95]***	0.86 [0.77, 0.96]**	0.84 [0.74, 0.95]**	0.94 [0.89, 0.99]*
Intervention-day shift	1.18 [0.83, 1.67]	1.37 [0.72, 2.62]	1.44 [0.65, 3.18]	0.94 [0.65, 1.36]
Post-intervention slope	1.00 [0.99, 1.02]	1.01 [0.97, 1.04]	0.97 [0.93, 1.02]	1.00 [0.97, 1.03]
*Covariates*				
Age	0.99 [0.97, 1.02]	0.99 [0.94, 1.03]	0.96 [0.91, 1.01]	0.99 [0.95, 1.02]
Gender (Male)	1.01 [0.53, 1.92]	0.58 [0.17, 1.91]	0.66 [0.18, 2.46]	1.80 [0.77, 4.21]
Education	0.90 [0.70, 1.18]	0.85 [0.53, 1.38]	0.81 [0.47, 1.38]	0.85 [0.60, 1.22]
Hispanic	0.38 [0.17, 0.87]*	0.25 [0.05, 1.17]	0.11 [0.02, 0.60]*	0.20 [0.07, 0.61]**
Financial status	1.22 [0.86, 1.72]	1.44 [0.76, 2.71]	1.39 [0.69, 2.81]	1.22 [0.76, 1.95]
AUDIT total	1.04 [1.00, 1.08]	1.01 [0.94, 1.09]	1.15 [1.06, 1.25]***	1.00 [0.95, 1.05]
Weekend	1.15 [0.95, 1.40]	1.07 [0.75, 1.54]	1.69 [1.09, 2.62]*	1.05 [0.85, 1.29]
*Random effects*				
Random intercept SD	0.81	1.45	1.55	0.92
Random post-intervention slope SD	—	—	—	0.05
NB dispersion (θ)	1.07	—	—	1.20
Random-slope LRT	χ^2^(1) = 0.00, p = 1.00	χ^2^(1) = 0.21, p = .647	χ^2^(1) = 0.00, p = 1.00	χ^2^(1) = 5.22, p = .022

*Note*. Drinks/day and planned drinks were modeled with a negative binomial (type 2) distribution and log link; any drinking and heavy drinking day were modeled with a Bernoulli distribution and logit link. IRR = incidence rate ratio; OR = odds ratio. NB dispersion (θ) is the negative binomial type 2 dispersion parameter. Continuous covariates were grand-mean centered.

* *p* < .05.

** *p* < .01.

*** *p* < .001.

**Table 3a:** A-path: Effects of the Intervention on Day-Level Importance and Confidence of Limiting Drinking (N = 33 participants, Observations = 799 person-days)

Predictor	Importance	Confidence
	*b (SE)*	*b (SE)*
*Time and intervention effects*		
Pre-intervention slope	0.00 (0.05)	0.14 (0.05)**
Intervention-day shift (path *a*)	0.73 (0.29)*	0.27 (0.32)
Post-intervention slope	0.00 (0.03)	−0.02 (0.03)
*Covariates*		
Age	0.04 (0.04)	0.02 (0.04)
Gender (Male)	−1.31 (1.09)	0.32 (1.01)
Education	−0.28 (0.44)	0.10 (0.41)
Hispanic	2.34 (1.39)	0.37 (1.28)
Financial status	−0.61 (0.59)	0.07 (0.55)
AUDIT total	0.18 (0.07)*	−0.02 (0.06)
Weekend	−0.08 (0.16)	0.46 (0.17)**
*Random effects*		
Random intercept SD	2.79	2.56
Random post-intervention slope SD (t2)	0.15	0.12
Residual SD	1.99	2.19
Random-slope LRT	χ^2^(1) = 77.86, p < .001	χ^2^(1) = 39.88, p < .001

*Note*. Values are unstandardized regression coefficients with standard errors in parentheses. Continuous covariates were grand-mean centered.

* *p* < .05.

** *p* < .01.

*** *p* < .001.

**Table 3b:** B-path and Adjusted Direct Effects of the Intervention on Daily Drinking Outcomes (N = 33 participants, Observations = 799 person-days)

Predictor	Drinks/day	Any drinking	Heavy drinking day	Planned drinks
	*IRR [95% CI]*	*OR [95% CI]*	*OR [95% CI]*	*IRR [95% CI]*
*Mediators*				
Importance (WP; path *b*)	0.92 [0.87, 0.97]**	0.83 [0.75, 0.93]***	0.78 [0.64, 0.96]*	0.83 [0.78, 0.89]***
Importance (BP)	0.97 [0.86, 1.09]	1.06 [0.84, 1.35]	1.25 [0.91, 1.70]	0.81 [0.70, 0.95]**
Confidence (WP; path *b*)	0.88 [0.83, 0.93]***	0.80 [0.71, 0.89]***	0.68 [0.60, 0.78]***	0.87 [0.81, 0.93]***
Confidence (BP)	0.81 [0.71, 0.92]**	0.62 [0.47, 0.81]***	0.52 [0.36, 0.74]***	0.83 [0.70, 0.97]*
*Adjusted intervention effects*				
Pre-intervention slope	0.92 [0.87, 0.97]**	0.89 [0.79, 1.00]	0.88 [0.76, 1.02]	0.95 [0.90, 1.00]*
Intervention-day level shift	1.28 [0.93, 1.76]	1.60 [0.79, 3.23]	1.92 [0.75, 4.90]	1.07 [0.77, 1.49]
Post-intervention slope	1.00 [0.99, 1.02]	1.00 [0.97, 1.04]	0.98 [0.93, 1.03]	1.01 [0.99, 1.03]
*Covariates*				
Age	1.00 [0.98, 1.02]	0.99 [0.95, 1.03]	0.96 [0.91, 1.01]	0.99 [0.97, 1.02]
Gender (Male)	1.04 [0.61, 1.78]	0.70 [0.23, 2.13]	1.03 [0.27, 3.98]	1.69 [0.85, 3.35]
Education	0.92 [0.74, 1.14]	0.89 [0.58, 1.38]	0.82 [0.48, 1.40]	0.83 [0.63, 1.10]
Hispanic	0.49 [0.24, 1.00]*	0.24 [0.05, 1.10]	0.07 [0.01, 0.44]**	0.35 [0.14, 0.90]*
Financial status	1.17 [0.87, 1.58]	1.43 [0.77, 2.64]	1.47 [0.69, 3.13]	1.08 [0.74, 1.58]
AUDIT total	1.04 [1.00, 1.08]*	1.00 [0.92, 1.08]	1.14 [1.04, 1.26]**	1.03 [0.98, 1.08]
Weekend	1.20 [1.00, 1.42]*	1.15 [0.78, 1.70]	2.26 [1.35, 3.80]**	1.04 [0.87, 1.25]
*Random effects*				
Random intercept SD	0.63	1.25	1.46	0.80
Random slope SD	0.05	<.001	0.33	0.09
Random slope SD	0.10	0.14	<.001	0.14
NB dispersion (θ)	1.15	—	—	1.45
Random-slope LRT vs. RIO	χ^2^(2) = 14.87, p < .001	χ^2^(2) = 2.04, p = .360	χ^2^(2) = 8.55, p = .014	χ^2^(2) = 35.46, p < .001

*Note*. WP = within-person, BP = between-person. Values are incidence rate ratios (IRRs) or odds ratios (ORs) with 95% confidence intervals.

* *p* < .05.

** *p* < .01.

*** *p* < .001.

**Table 3c:** Indirect-Effect Estimates of the Intervention on Daily Drinking Outcomes via Day-Level Importance and Confidence of Limiting Drinking (N = 33 participants, Observations = 799 person-days)

Mediators	Indirect path	Drinks/day	Any drinking	Heavy drinking day	Planned drinks
		*Indirect [95% CI]*	*Indirect [95% CI]*	*Indirect [95% CI]*	*Indirect [95% CI]*
Importance	*a* × *b*	−0.064 [−0.138, −0.011]*	−0.132 [−0.282, −0.023]*	−0.181 [−0.430, −0.012]*	−0.136 [−0.260, −0.029]**
Confidence	*a* × *b*	−0.034 [−0.124, 0.046]	−0.061 [−0.222, 0.082]	−0.101 [−0.353, 0.137]	−0.038 [−0.139, 0.050]

Note.

* *p* < .05.

** *p* < .01.

*** *p* < .001.
